# Microbiota and chronic inflammatory arthritis: an interwoven link

**DOI:** 10.1186/s12967-016-0989-3

**Published:** 2016-08-04

**Authors:** Andrea Picchianti Diamanti, M. Manuela Rosado, Bruno Laganà, Raffaele D’Amelio

**Affiliations:** 1 Department of Clinical and Molecular Sciences, II School of Medicine, S. Andrea University Hospital, “Sapienza” University of Rome, Rome, Italy; 2 Consultant in Immunology, Rome, Italy; 3 Department of Clinical and Molecular Medicine, School of Medicine and Psychology, S. Andrea University Hospital, “Sapienza” University, Via di Grottarossa 1039, 00189 Rome, Italy

**Keywords:** Microbiota, Chronic inflammatory arthritis, Immunosuppressant, Probiotic, Stress, Diet, Smoke

## Abstract

**Background:**

Only recently, the scientific community gained insights on the importance of the intestinal resident flora for the host’s health and disease. Gut microbiota in fact plays a crucial role in modulating innate and acquired immune responses and thus interferes with the fragile balance inflammation versus tolerance.

**Main body:**

Correlations between gut bacteria composition and the severity of inflammation have been studied in inflammatory bowel diseases. More recently similar alterations in the gut microbiota have been reported in patients with spondyloarthritis, whereas in rheumatoid arthritis an accumulating body of evidence evokes a pathogenic role for the altered oral microbiota in disease development and course. In the context of dysbiosis it is also important to remember that different environmental factors like stress, smoke and dietary components can induce strong bacterial changes and consequent exposure of the intestinal epithelium to a variety of different metabolites, many of which have an unknown function. In this perspective, and in complex disorders like autoimmune diseases, not only the genetic makeup, sex and immunologic context of the individual but also the structure of his microbial community should be taken into account.

**Conclusions:**

Here we provide a review of the role of the microbiota in the onset, severity and progression of chronic inflammatory arthritis as well as its impact on the therapeutic management of these patients. Furthermore we point-out the complex interwoven link between gut-joint-brain and immune system by reviewing the most recent data on the literature on the importance of environmental factors such as diet, smoke and stress.

## Background

Recent advances in sequencing technologies have allowed the deep characterization of the human microbiota, thus greatly improving our knowledge on the role of the microbiome in human health and disease. Human microbiome project consortium studies [1] demonstrated that healthy individuals have not only a high degree of bacterial diversity, dependent on their habitat (intestine, oral cavity, skin or vagina), but that there is also a remarkable inter-individual variability at the level of species. In spite of the large amount of different species found at different sites, at the level of microbial community there is evidence for a certain constancy that preserves both the function and the bacterial gene profiling associated to specific tissue sites. For example the anaerobic firmicutes/bacteroidetes spp. dominate the intestine whereas actinobacteria and proteobacteria spp. are highly abundant in the skin. Given the strong variability and abundance of microbes living in close relation with us, it becomes a difficult task to define what should be considered the “normal” microbiome. Moreover, the same microbe may behave as commensal or as pathogen depending on the dietary components, nutritional milieu, co-infection or genetic background of its host. Albeit, it is still generally accepted that commensal bacteria contribute to immune homeostasis, whereas immune reaction against intestinal flora is accepted as a pathological sign. This distinction, however, is not absolute, because it is possible to find in serum of healthy subjects antibodies specific to commensals as well as circulating T cells able to react against non-pathogenic bacteria [2, 3]. Gut colonization, at birth, is indispensable to trigger and endure maturation of the mucosal immune system. At this phase, in which the immune structures are not yet fully developed, microbiota plays a crucial role in setting-up innate and acquired immune responses and thus interferes with the fragile balance among inflammation, infection and tolerance that may be “imprinted” for life. It is known that germ-free mice have hypoplastic Payer’s patches (PP), lack isolated lymphoid follicles (ILF) in the intestine and have low levels of IgM natural antibodies in the serum and that this phenotype can be reverted by gut colonization [4]. Undoubtedly, colonization of the distal small intestine by segmented filamentous bacteria (SFB) is crucial for the development of resident lamina propria dendritic cells to secrete IL-6 and IL-22 triggering the loop of Th_17_-T regulatory (Treg) cell [5] generation in the new-born gut. The discovery of a link between defined members of the microbiota and the induction of Treg cells generated a huge interest. However, very recent studies demonstrate that the homeostasis of the colonic Treg compartment relies more on the synergistic effect of different bacterial strains than on a single individual species of that strain. This observation strongly suggests the importance of the bacterial community in creating a microenvironment able to sustain the generation and maintenance of the anti-inflammatory milieu [6, 7]. Another player, particularly important in the development of arthritis that depends upon SFB colonization, is the follicular T helper cell (Tfh). Tfh cells are a T cell subset specialized in providing T cell help to B cells in the context of germinal center (GC) formation. By inducing somatic hypermutation and class switching Tfh cells promote the generation of high affinity antigen specific memory B cells as well as antibody producing cells [8, 9]. Several studies have recently shown, in a K/BxN RA mouse model [10], that SFB interferes with Tfh differentiation in Payer’s Patches and supports antigen specific Tfh dissemination at systemic sites resulting at increased autoantibody production [11, 12].

As mentioned before not only bacteria but also bacterial-derived products can directly or indirectly control the function of epithelial and inflammatory cells and thus being key factors in initiating/maintaining disease. For instance, recognition of the metabolite short-chain fatty acids (SCFAs) by innate immune cells is critical for the regulation of inflammation in response not only to intestinal injury, but also in arthritis and allergy [13]. Commensals can also tune the inflammatory potential of monocytes by promoting the production of the lipid mediator PGE2 that, in turn, reduces activation of tissue-damaging neutrophils [14]. In addition to the intestinal microbiota with its regulatory elements, studies describing the microorganism composition of the oral cavity have also been undertaken and will soon give new insights on human health and disease. The oral mucosa contains several “ecological” niches that are able to harbor a large variety of bacteria, viruses and fungi. Although the oral microbiome may be influenced by diet, oral hygiene and smoke, a recent study showed that oral microbiome of rheumatoid arthritis (RA) individuals correlates with patient clinical features and differs from microbiome of healthy controls [15]. In conclusion, significant efforts have been recently undertaken to better understand our microbiome in terms of composition and metabolic pathways aiming at using microbes or microbial products for targeted therapy in various immunological dysfunctions, including autoimmunity.

### Microbioma and host genetic background

Host genetic background, and in particular major histocompatibility complex (MHC) genes, plays a pivotal role in the microbial composition of the gut [16]. In this context, to the previously genome-wide studies in which association between HLA genes and susceptibility to autoimmunity has been demonstrated, the microbiome as potential environmental factor causing disease should now be inserted. It is known that certain HLA class-II alleles, such as the sequence 60-74 in the third hypervariable region 3 of DRB1*0401 gene, are associated with increased susceptibility to RA, not only caused by abnormal CD4 T cell activation, but also due to the generation of autoantibodies. In this case, infectious agents trigger the production of antibodies against self-antigens through a mechanism called molecular mimicry or cross-reactivity [17]. Another example is given by the HLA B27, HLA with strong genetic risk factor for the pathogenesis of spondyloarthritis (SpA), that has an amino acid sequence shared with a nitrogenase from Klebsiella and other proteins present in Gram-negative bacteria. In this case, antigen mimicry induces cross-reactivity between microbial epitopes and self-proteins resulting in autoreactive immune responses. The contribution of antigen mimicry in promoting autoimmune disease has been confirmed in rodents expressing human HLA B27 molecules that are preserved from developing peripheral arthritis, similar to SpA disease, when raised in a germ-free environment [18, 19]. Although pathogenic bacteria are clearly involved in promoting autoimmune diseases also nonpathogenic commensal bacteria may play a role through the chronic activation of the innate and acquired immune responses [20]. Worth to mention that often in rheumatic diseases, patients may present leakage of the mucosal barrier or altered immune functions strongly conditioning the persistence of antigenic stimulation even at articular sites [21]. In the context of altered immune functions, Toll like receptors (TLR) by modulating the binding to bacterial products, interfere with Th17 generation and inflammation and thus may contribute to the persistence of RA disease [22]. Notwithstanding TLR polymorphisms were shown not to be directly involved in RA pathogenesis [23, 24], its role, not only in regulating innate immune responses, but also in the epithelial cell homeostasis at mucosal sites, may upon environmental exposure become a pathogenic factor. Another important class of receptors linked to bacterial sensing are G-couple protein receptors family (GPR). Several members of this family are orphan receptors binding several ligands including bacterial products and dietary metabolites derived from gut microbiota. The receptor with the strongest link with bacterial sensing is the caspase recruitment domain-containing 9 (CARD9). This receptor is strongly associated with disease in Crohn’s patients, and although CARD9 polymorphisms resulting in reduced CARD9 expression have been identified in AS, further studies are needed to sustain the association between CARD9 and AS disease [25, 26].

### Microbiota and spondyloarthritis

SpA are chronic progressively disabling inflammatory diseases with a reported prevalence of 2.4/1000 and with a number of estimate patients of 1.5 × 10^6^ only in Europe [27]. The SpA group includes ankylosing spondylitis (AS), psoriatic arthritis (PsA), reactive arthritis, SpA associated to inflammatory bowel disease (IBD) (also known as enteropathic arthritis), acute anterior uveitis and the most recently acquainted axial non radiographic SpA. Since their pleomorphic phenotype SpA early diagnosis and management is often a challenge for the clinician. In fact, SpA patients may come to the healthcare units with an axial involvement (i.e. sacroiliitis) and/or peripheral arthritis frequently associated to extra-articular manifestations affecting skin, eyes, and intestine. In particular SpA shares various clinical, genetic and immunological features with IBD and, although enteropathic SpA represents the clinical evidence of association between the two, subclinical intestinal abnormalities can be found up to 50 % of SpA patients [28]. Translocation of intestinal microbes, caused by the increased permeability of the intestinal epithelial cell layer, or increased exposure to microbial products are two possible links connecting gut dysbiosis and joint pathology [29]. In recent years different authors have reported abnormalities in the gut microbiota in patients with IBD, suggesting that these diseases can result from altered interactions between gut microbes and the mucosal immune system [30–32]. Although, gut microorganism composition plays an important role in chronic inflammatory disorders, no specific bacterial strain has been yet identified as instrumental cause of the disease. Since SpA is frequently associated to IBD, it is coherent to hypothesize a potential role for the gut microbiota and consequently for dysbiosis also in SpA pathogenesis. Indeed, the first experimental evidence for this link aroused from the animal model of human AS [33]. The ankylosing enthesopathy (ANKENT) mouse which is spared from developing joint ankylosis in germfree environment develops a less severe form of the disease in pathogen-free conditions [34]. Another study, in germfree HLAB27/human β2 microglobulin transgenic rats using bacterial transfers, revealed that only the gut colonization with bacteria isolated from the intestinal flora of Crohn’s disease patients was able to induce bowel and joint inflammatory disease [35]. More recently, Rosenbaum et al. [19] using high throughput 16S sequencing techniques studied the microbial composition present in the cecum of these transgenic rats and found a significant expansion of *Prevotella* and *B. vulgatus* spp. In contrast to IBD there are few data on the gut microbiota composition and function in SpA patients. Stebbings et al. are the only group that reported two comparative studies on the microbiota isolated from fecal samples of AS patients and healthy controls. In the first study, they found a higher proportion of sulphate-reducing bacteria in the fecal samples from AS patients with no significant difference in the microbiota composition using denaturing gradient gel electrophoresis [36]. In the second study, using an indirect in vitro experimental approach, they observed that IL-10 production by peripheral blood mononuclear cells (PBMC) measured in the supernatants of the cell cultured with autologous *Bacteroidetes* were lower in the AS group compared with controls suggesting that patients have a reduced ability to generate tolerogenic environment in their gut [37]. Stool and blood specimens collected from children with enthesitis-related arthritis showed less *Faecalibacterium prausnitzii* spp. and *Lachnospiraceae* family members and a significant increase in *Bifidobacterium genus*; the authors found also differences in the humoral responses mainly altered levels of the specific IgA antibodies reacting against the aforementioned bacteria. This suggests that the imbalance observed in the bacterial community may contribute to disease onset [38]. Another place heavily exposed to microorganisms, where uncontrolled inflammation may lead to autoimmunity, is the skin. Recent studies, using different sampling techniques, such as skin swabs or punch biopsies, have both found a significant reduction in *Actinobacteria* and *Propionibacteria* in skin lesions of patients with psoriasis and an increase in *Firmicutes* as compared to healthy dermis [39, 40]. PsA can be associated with both psoriasis and IBD, suggesting an etiologic overlap between the joint inflammation and both gut and skin pathology in which the microbiome may be the common mediator. The group of Scher [41] showed very recently a reduced diversity in the gut microbiota composition of patients with PsA and skin psoriasis when compared to healthy controls. More specifically, this study reports a significant reduction in members of the bacterial genus *Akkermansia, Ruminococcus,* and *Pseudobutyrivibrio* in feacal samples from PsA patients.

### Microbiota and rheumatoid arthritis

RA is an inflammatory autoimmune disease potentially leading to functional disability, with about 30 % of patients unable to work after 3 years of disease. It is characterized by the positivity of specific autoantibodies [rheumatoid factor (RF) and anti-citrullinated protein antibodies (ACPAs)] and the presence of an erosive simmetric synovitis affecting predominantly small joints such as hands, wrists and feet. There is strong evidence that the highest joint inflammation level may be observed early (75 % erosions within the first 2 years) and that timely treatment is able to slow down the progression of bone and join damage measured by radiographic scanning. This awareness of the need of early intervention, joined to the availability of more effective methods for early diagnosis/prognosis and the development of new therapeutic tools, pushed clinicians to introduce and characterize the concept of “early rheumatoid arthritis” (eRA). A comparative study conducted on fecal samples of patients with newly diagnosed RA and of patients with fibromyalgia showed that the first group had significantly lower abundance of common commensals including *Bifidobacteria* and *Bacteroides* [42]. Another study investigated the composition of fecal *Lactobacillus* communities in eRA patients and found a higher diversity of bacteria than in healthy controls [43]. On the other hand Chen et al. have recently reported a decreased gut microbial diversity in RA patients, which correlated with disease duration and autoantibody levels. In fact, analysis of the 16S ribosomal DNA of fecal samples from RA patients showed the expansion of rare taxa that allowed authors to develop a prediction model identifying three genera, *Collinsella, Eggerthella,* and *Faecalibacterium* segregated with RA disease [44].

Scher et al. performed 16S sequencing on 114 stool samples from eRA patients and controls, and shotgun sequencing on a subset of all the samples. Results showed an abundance of *Prevotellacopri* spp. with concomitant loss of various *Bacteroidetesphylum* members in the stool of RA patients [45]. The inoculation of fecal samples from eRA patients into germ-free arthritis-prone SKG mice showed increased sensitivity to arthritis via activation of autoreactive T cells in the intestine. T cell activation in these mice was most probably caused by dysbiosis. In fact, deep sequencing of the 16S rRNA of fecal eRA samples showed a microbiota markedly dominated by *Prevotellaceae* [46].

Beyond gut microbiome, an accumulating body of evidence strongly evokes a pathogenic role for the altered oral microbiota in RA development and disease course. Interestingly Zhang et al. using metagenomic shotgun sequencing and a metagenome-wide association study (MGWAS) found a concordant dysbiosis of both fecal and oral samples from RA patients that was partially resolved after RA treatment. In fact, microbiome changes correlated with clinical measures in particular, *Haemophilus* spp. was depleted and negatively correlated with levels of serum autoantibodies, whereas *Lactobacillus salivarius* was over-represented. This was reinforced by the observation that *Lactobacillus salivarius* was also present in increased amounts in very active RA disease [15].

The oral healthy microbiome is second only to the gut in total microbial species and is extensively represented by the bacteria *phyla Firmicutes, Bacteroidetes, Proteobacteria, Actoniobacteria, Spirochaetes* and *Fusobacteria* [47]. The periodontitis (PD)-associated bacteria are widely studied and *Porphyromonas gingivalis, Tannerella forsythia, and Treponema denticola* are the species most frequently associated with this chronic inflammatory oral disease [48, 49]. RA and the most severe forms of PD share several immunological and morphological features, such as increased levels of pro-inflammatory cytokines and metalloproteinases that can lead to several tissue and bone alterations [43, 50]. Different groups have joined efforts in studying the epidemiological association between these two diseases aiming at identifying a possible pathogenic link. The most convincing hypothesis is that certain species of bacteria present in the oral cavity induce break of tolerance, by exerting an abnormal citrullination activity. Citrullination is a post-translational reaction consisting of the conversion of arginine into citrulline, which can have important consequences for the protein structure and function. Citrullinated proteins arise by the activity of peptidyl-arginine-deiminases (PAD) enzymes that catalyze the modifications of peptidyl-arginine to peptidyl-citrulline on different self-proteins, among which α-enolase, keratin, fibrinogen, fibronectin, collagen, and vimentin; loss of tolerance to such neo-epitopes elicits an ACPAs response that may result into disease [51]. In 2004 Rosenstein et al. suggested that the humoral response to *P. gingivalis* could provide a trigger for the development of RA [52]. *Porphyromonas gingivalis* is actually the only known bacterium carrying a PAD enzyme [53]. Although PAD enzymes expressed in *P. gingivalis* are quite different from the human variants it has been reported that these bacterial enzymes can lead to irreversible citrullinated peptides, mainly in α-enolase and fibrinogen, in RA patients [54, 55]. The importance of oral bacteria as a disease-causing element by this mechanism has been corroborated by a number of studies showing strong correlations between increased prevalence of periodontitis and severity in RA, as well as, by the detection of anaerobes and high antibody titers against periodontal bacteria both in the serum and in the synovial fluid of RA patients [45, 56, 57]. A recent paper from Scher et al. by using a 16S rRNA pyrosequencing approach, showed that severe PD is already present at the onset of RA in newly diagnosed and untreated patients. Although Scher demonstrated that RA patients have twice the amount of *P. gingivalis* as compared to healthy controls this observation did not correlate with the presence of APCA [58]. Lange et al. have recently demonstrated that children with CCP-positive Juvenile Idiopathic Arthritis (JIA) have higher antibody responses to *P. gingivalis* and poor oral health than children with APCA-negative JIA [59].

In a murine model of chronic Ag-induced arthritis (AIA) Queiroz-Junior et al. showed that PD resulted simultaneously in synovial joint hyperplasia, severe alveolar bone loss, migration of osteoclasts and release of pro-inflammatory cytokines; moreover anti–TNF-α therapy was able to prevent the development of both AIA and PD [60]. Two other groups evaluated the role of the conventional therapy for PD and root planning in improving RA inflammation and activity suggesting a link between PD and RA. In the first study the authors showed a better outcome in RA patients who received a PD treatment in addition to standard disease modifying antirheumatic drugs (DMARDs) and anti-TNF-α [61]. The second study reported the presence of PD in RA patients as a prognostic factor associated to a poor response to anti-TNF-α therapy [62].

Finally, the first case of a complete and long-lasting recovery after periodontal treatment, in a male patient with newly onset of RA and PD has been recently reported, suggesting that in selected early RA cases, prompt periodontal infection treatment may induce disease regression, thus avoiding the development of a chronic and progressive arthritis [63].

### Environmental factors influencing arthritis and microbiota

#### Smoke

Environmental factors that influence periodontal bacterial community may represent risk factors for the development of autoimmune diseases. One of the most well studied risk factors affecting both periodontal disease and arthritis is smoke [64, 65]. Cigarette smoke can lead to several immune dysregulations, such as modulation of apoptosis with the increased exposure of sequestered intracellular autoantigens and increased release of free radicals that can cause mutations or gene activation [66, 67]. It can also induce alterations of T cell function, impair humoral responses and change the balance between regulatory and inflammatory cytokines [66, 68–70]. Onozaki et al. incubated human synovial fibroblasts with 5 % cigarette smoke extract and by using microarray and real time PCR observed a significant upregulation of the heat shock proteins DnaJA4, DnaJB4, DnaJC6, HspB8 and Hsp70 that have been shown to lead the activation of inflammatory signaling pathways in RA [71]. In RA patients, cigarette smoke can act via the induced production of ACPAs. The smoke attributable risk for RA is variable; a large prospective study reports a risk ratio ranging from 2.6 to 3.8 in former and heavy smokers compared with non smokers [72]. The association is stronger in men, in patients positive for ACPAs and expressing the shared HLA epitope (HLA-DRB1) [73, 74]. Data on tobacco consumption and RA disease severity are contrasting, however there is agreement on the increase in extra-articular manifestations and earlier onset of disease in RA smokers as compared to non-smokers [75]. Of note, different authors and registries data highlighted a significant lower response to synthetic and biological (anti-TNF-α) drugs, as well as, a reduction in the probability of reaching disease remission in RA smokers [76–78]. The negative impact of tobacco on drug response can be also related to its effect on the drug metabolic rate and consequent pharmacokinetics alteration as previously reported [79]. In PD, the effect of smoke on microbiota are controversial, however, it has been demonstrated that *P. gingivalis, Tannerella forsythia, Aggregatibacter actinomycetemcomitans* spp. and, more recently, *Parvimonas* and *Treponema* have a higher prevalence on the oral cavity of smokers than in non-smokers subjects [80, 81]. Few are the studies that have analysed the influence of smoking on the composition of the gut microbiota. However Biedermann et al. have recently showed significant changes after smoking cessation with an increase in *Firmicutes* and *Actinobacteria* accompanied by a reduction of *Bacteroidetes* [82].

#### Diet

Diet is another modifiable environmental factor that can influence both microbiota composition/diversity and arthritis onset/outcome. A certain cause-effect relation between diet and arthritis is hard to perform thus genetics and living conditions cannot be ruled out as important interfering factors. However the relevance of diet composition is also suggested by the significant differences in the human microbiota structure between geographically distant populations [83, 84]. It has been reported that a diet rich in animal proteins, simple sugars and saturated fats, all typical components of food intake in western countries, is characterized by a less diverse microbiome associated with the *Bacteroidetes enterotype*, whereas *Prevotella* is the most prevalent in people assuming high fiber diets rich in fruits and vegetables [85]. The fecal microbiota of African children (characterized by a diet prominently composed of plant-derived carbohydrates) is enriched in *Prevotella* and *Xylanibacter* and depleted in *Bacteroides* compared to the European and USA children [86]. Recent data also demonstrated that dietary intervention can modify, even rapidly, gut microbiota structure and overwhelms inter-individual differences in microbial gene expression. Subjects replacing a diet high in fibers with a short term one (ad libitum for 5 days) rich in animal-based high fat and proteins have an increase in bile-tolerant microorganisms (*Alistipes, Bilophila,* and *Bacteroides phyla*) and a decrease in *Firmicutes*, such as *Roseburia, Eubacterium rectale* and *Ruminococcus bromii* that metabolize dietary plant polysaccharides [87]. However, recently, Cotillard et al. demonstrated that dietary intervention, such as a change to a low caloric diet, is able to increase microbiome gene richness only in overweight subjects characterized by low gene richness at baseline, suggesting that the responses to nutritional modulation are influenced by the initial microbiota structure present in a given individual [88]. A 2009 Cochrane review evaluated the effects of dietary interventions in RA patients which included fourteen randomized clinical trials (RCTs) and one case control study, with a total of 837 patients [89]. Among these RCTs, one found that fasting, followed by 13 months on a vegetarian diet, can reduce pain and morning stiffness but was unable to restore the physical function with respect to control group [90]. Another RCT found that a 12-week Cretan Mediterranean diet could reduce pain but was unable to rescue physical function or morning stiffness [91]. Finally, two other RCTs compared 4 weeks elemental diet with an ordinary diet and reported no significant differences in pain, function or stiffness [92, 93]. Based on these observations the authors of the Cochrane concluded that favorable disease outcomes given by any dietary manipulation, including vegetarian, mediterranean, elemental and elimination diets, on RA patients are not conclusive. The uncertainty related to the validity of such results is due to the poor quality of the reported data and also to the possible adverse events related to severe diet restriction. As alternative to full diet change, diet supplementation with derivatives of polyunsaturated fatty acid (PUFA) omega-6 has also been investigated. Arachidonic acid (ARA), a product of PUFA-omega-6, is a substrate for the synthesis of important mediators of inflammation called eicosanoids which are known to participate in the pathogenesis of chronic joints synovitis in RA and SpA and are commonly target by NSAIDs [94]. Other PUFAs with similar beneficial characteristics are the omega-3 eicosapentaenoic acid (EPA) and docosahexaenoic acid (DHA) that are present in seafood, especially oily fish, and in fish oil-type supplements [95]. These fatty acids are involved in ARA availability/metabolism and influence several immuno-inflammatory responses such as dendritic and T cell function, the production of inflammatory cytokines and reactive oxygen species in the health and in RA patients [96–100]. In western country diet generally contains much more omega-6 than to omega-3 PUFA probably due to excessive intake of processed vegetable oils. In mouse models of arthritis a diet rich in fish omega-3 oils was able to delay disease onset and reduce joint synovitis compared to an omega-6 based diet [101, 102]. Several authors evaluated the effect of such a dietary composition in patients with RA with contrasting results, however a recent meta analysis including selected published studies between 1985 and 2009 has reported a moderate role for supplementation with omega-3 fatty acids in decreasing joint pain and morning stiffness [95]. Vitamin D is another dietary nutrient that has demonstrated increasing relevance in reducing inflammatory autoimmune diseases in the last years. Vitamin D levels are influenced by several factors such as sun exposure, age, ethnicity, body mass index, use of medications and supplements. It is known that vitamin D can reduce inflammation and modulate the immune system and an association between vitamin D serum levels and rheumatoid disease in mouse models of arthritis has been reported [103, 104]. Furthermore, the prevalence of RA is higher in areas where vitamin D deficiency is present, however data regarding vitamin D supplementation in RA patients are insufficient and contrasting [105, 106].

#### Stress

Stress consists in a stimulus that precipitates a reaction in the brain with the consequent activation of physiological fight-or-flight responses [107, 108]. This reaction can modify hypothalamic–pituitary-adrenal (HPA) axis by the secretion of hormones like gluco-corticoid, mineral-corticoid, epinephrine and norepinephrine that are able to modulate the immune system by interfering with cytokine secretion and immune cell redistribution. The impact of stress on immune function has been extensively investigated in healthy subjects exposed to different type of stress factors (eustress vs distress), intensity (minor stress such as work stress and interpersonal relations, or major stress like death or severe illness of a parent) and duration (acute vs chronic stress) [109–111]. Stress can modulate the immune system by inducing a shift away from cellular immunity (T helper type 1, Th1) towards humoral responses (T helper type 2, Th2) [112, 113]. The effect of stress can be different according to the type of stressor, subject and environment, however it is generally accepted that short-term stress can enhance innate and adaptive immune responses, whereas chronic or long-term stress can induce immunosuppression by decreasing immune cell numbers and function and/or increasing active immunosuppressive mechanisms [114, 115]. Since stress has the ability of modulating immune function and the empirical observations that stress could precede autoimmune onset and disease flares, different authors evaluated the impact of stress in these pathologies [116–118]. A proposed mechanism for the brain-immune axis to influence disease activity is the induction of pro-inflammatory cytokine secretion; indeed an increase in IL-6 and IL-1 has been observed during acute stress in patients with autoimmune disorders [112, 119–121]. Stress can also be an autoimmune inducer by influencing lymphocyte number and activity. It has been reported, in fact, that an acute stress in healthy subjects is able to induce an imbalance between peripheral effector T cells (CD8^pos^CCR7^neg^) and Tregs (CD4^pos^FOXP3^pos^) in favor of the effector arm of the immune system [114]. Despite these observations, strong evidences for a clinical role of stress in RA onset/flares are still a matter of controversy. Different authors in fact found no significant association between exposure to previous stressful events and increased incidence of RA [122, 123], furthermore, in most of the studies no significant correlation between disease severity and major stressors has been found [119, 124]. In contrast to the studies mentioned above, more recently, Zautra et al. have reported an increased incidence of disease flare up in RA women exposed to minor stress [125] and Potter’s group described a worse disease presentation and radiographic outcome in a 5-year follow-up study of RA patients exposed to high levels of daily minor stress [126]. The axis between brain and immune system involves also the gut microbiome. The complex network between gut microbiota and the brain encompasses the central and autonomic nervous system and communicates via hormonal neural cytokine pathways. In recent years there is a growing interest in elucidating this network and in finding correlations between microbiota composition/diversity and stress related disturbances such as depression anxiety and pain. The first evidence comes from several experiments on stress induction in GF mouse models that reported a reduction of the anxiety-like behaviors in GF mice as compared to specific pathogen free mice; furthermore this behavioral condition was confirmed by changes in plasticity-related genes in the hippocampus and amygdala [127]. Always in mice, Nishino et al. showed that the type of commensal microbiota present in the gut can influence behavior by describing that the monoassociation with *Blautia coccoides* spp. in GF mice reduced anxiety-like behavior in open field, whereas monoassociation with *Bifidobacterium infantis* reduced activity without affecting anxiety [128]. Another group using a mouse model of induced anxiety and depression via olfactory bulbectomy, reported alterations in gut microbiota, which were related to the activation of the hypothalamic pituitary adrenal axis [129]. Finally last year Topol et al. showed in rats a reduction in the total number of Xbp1^pos^ lymphocytes in the gut associated lymphoid tissue of the ileum in animals under induced chronic social stress suggesting a role for Xbp1 expression levels as a trigger of inflammation and thus as potential link between stress and autoimmunity [130]. For what concerns depression and its interplay with gut flora few relevant data is available. Only, recently Naseribafrouei addressed this topic and demonstrated an overrepresentation of *Bacteroidetes* spp. and a reduction of *Lachnospiraceae* in the fecal microbiota of 37 patients with depression [131]. In individuals suffering of irritable bowel syndrome, in which anxious/depressed status plays an important role, it was described a reduction in *Bacteroidetes* and an increase in *Firmicutes phylum* representation [132–134]. Behavior modifications, induced by the probiotic mix, were reflecting the reduction in microglial activation and cerebral monocyte infiltration as well as the decrease in circulating TNF-α levels [135]. Despite these efforts studies that put together microbiota, nervous system and chronic inflammatory diseases in clear correlation of cause effect are still lacking.

### Therapy, microbiota and arthritis

Probiotics are defined as living organisms that if ingested in adequate amount can confer a health benefit to the host [136, 137]; lactic acid and bifidobacteria are the most frequently used types of bacteria. Probiotic use has been recently analysed in animal models of arthritis whereas few studies have been conducted in humans. First evidence for beneficial effects comes from two papers by So et al. in which they studied the outcome of *L. casei* (5 × 10^9^ CFU × 3 times per week) administration in CIA rats [138, 139]. Probiotic oral assumption produced a reduction in inflammatory (i.e. IL-1α, IL-6, IL-12, IL-1 and TNF-α) and an increase in regulatory cytokines (IL-10) accompanied by an improvement in disease signs, such as paw swelling and cartilage tissue damage. These results were confirmed by a more recent study, in which assumption of *L. casei* (2 × 10^8^ CFU/mL daily) demonstrated to exert significant protection in a CIA rat model by preventing synovial infiltration, pannus formation, and bone destruction compared to control rats and CIA rats treated with indomethacin [140]. In RA patients, three RCTs have been recently published with contrasting results. The first study evaluated the effects of the oral administration of a probiotic containing *Bacillus coagulans* (2 billion CFU) plus green tea extract, methy-sulfonyl-methane, vitamins and minerals versus placebo on 45 RA patients during a 2 months follow-up [141]. Patients receiving probiotic treatment resulted in a greater improvement in their global and self assessed disability as well as a greater reduction in serum C reactive protein (CRP) tender and swollen joints count. In 2011 Pineda et al. examined the effect of probiotic supplementation in 15 patients with established moderate RA under DMARDs, 14 matched RA patients were used as controls [142]. Patients received capsule containing either 2-billion CFU *Lactobacillus rhamnosus* and *Lactobacillus reuleri* or capsule without bacteria for a follow-up period of 3 months. At the end of the study there was no significant difference between treated and non-treated individuals in terms of clinical disease activity scores or in the main inflammatory serological parameters and cytokines. The last trial conducted by an Iranian group tested the effect of *L. casei* on 22 women with established RA as adjunctive therapy to the ongoing stable synthetic immunosuppressant in comparison to 24 women with RA that were used as control group [143]. Authors described a reduction in the swollen and tender joints count, as well as, in the patient global health and CRP at 3 months only in the group receiving *L. casei* (capsule containing a minimum of 108 CFU). Moreover, more patients in the probiotic group were found to have moderate response to the treatment and a reduction of serum levels of inflammatory cytokines compared to the placebo group. However, looking in depth, the beneficial effects given by probiotics appear less solid; in fact one should take into account that patients at baseline had a mean number of swollen and tender joints of zero and a normal CRP reflected by a mean disease activity score on 28 joints (DAS28) of 2.6 that is a value in which patients are already considered at clinical remission. Interestingly in 2009 one pilot study reported a significant reduction of bath ankylosing disease activity index (BASDAI) in patients with active SpA associated to quiescent ulcerative colitis (UC) after treatment with a probiotic regimen of *Lactobacillus acidophilus* and *Lactobacillus salivarius* [144]. However more recently a RCT developed by Jenks et al. involving 63 patients with active SpA receiving either a probiotic containing at least 10^8^ CFU of *Streptococcus salivarius, Bifidobacterium* and *Lactobacillus acidofilus* or placebo over a period of 3 months, failed to demonstrate significant differences in any of the clinical disease activity or functional domain such as BASDAI, bath ankylosing functional index (BASFI) and Maastricht spondylitis enthesitis score (MASES) between the 2 groups [145]. Of note, Bernston et al. have recently reported a case of a patient with severe JIA that a significantly improved his clinical status after several months of therapy with solely enteral nutrition. Patient also showed changes in his gut microbiome and metabolome [146].

There is a growing awareness that different drugs such as antibiotic and immunosuppressant can both influence and to be influenced by microbiota diversity and composition. Although the impact of antibiotics on microbiome is mostly temporary, their frequent use or administration during early infancy can exert permanent effects only partially understood. It is known that anti-cancer therapy frequently used also in treating chronic inflammatory arthritis, such as methotrexate and cyclophosphamide, changes dramatically the gut microflora. After these treatments often occurs a general depletion of the gut microbiota, the drop in bacteria diversity is accompanied by a reduction in the commensal anaerobic species in favor of potential pathogens that can lead to a deterioration of the gut barrier, altering epithelial cell permeability with consequently bacterial translocation [147]. The hypothesis that the gut microbiota can interfere with the outcome of anticancer therapy is not new, but only recently two independent groups have addressed this issue using mouse models. Iida et al. [148] showed that CpG-oligonucleotide administration in antibiotic-treated or in GF mice resulted in an impaired cytokine production by tumor-infiltrating myeloid-derived cells; furthermore these mice presented a deficient production of reactive oxygen species and cytotoxicity after chemotherapy. The other group [149] demonstrated that antibiotic-treated mice or GF were resistant to cyclophosphamide depleting action probably because of a reduction in the generation of Th17 cells, suggesting that an intact microbiota is necessary for successful tumor control in response to therapy. Sulphasalazine (SSA) is a drug commonly used both in IBD and chronic inflammatory arthritis such as SpA and RA. Different authors have analysed the role of the intestinal microbiota in SSA metabolism and found a reduction of SSA anti-inflammatory function in patients with UC previously treated with severe antibiotic regimens [150, 151]. In contrast to these observations, Lee et al. [152] did not find significant changes in plasma and urinary levels of SSA and of its metabolites in a small group of RA patients in whom a multi-strain probiotic regimen was administered. The impact of microbiota on the therapy outcome using biologics has not been extensively explored. A pioneer study addressing this issue focused at the analysis of fecal samples isolated from 33 Crohn Disease (CD) patients treated with anti-TNF-α aiming to identify, based on the gut microbiota composition, a factor predictive of relapse. They found a lower rate of bacterial species belonging to the *Firmicutes* phylum in relapse as compared to non relapse patients, and a consistent drop in *F. Prausnitzii* spp. that was able to predict the incidence of disease flares [153].

## Conclusions

In the last years there is a growing awareness regarding the role of the microbiota in the onset and outcome of patients with SpA and RA. Recently a reduction in the gut microflora diversity and alterations in its composition, similar to that observed in IBD, have been reported in patients with PsA; the increased permeability of the intestinal epithelial cell layer as well as the increased exposure to microbial products has been proposed as a mechanism that can connect dysbiosis to joint inflammation. In RA patients the most supported association between disease and microbiota is with the oral dysbiosis usually observed in patients with periodontitis. In particular, the presence of *P. gingivalis* could provide a trigger for RA onset in susceptible individuals by leading to the irreversible generation of citrullinated peptides.

To better understand the gut-joint link it is important to consider that several environmental factors such as diet, smoking and stress can influence both the microbiota diversity/composition and the arthritis onset/outcome. The relevance of diet is suggested by the differences in the gut microbiota between geographically and life style distant populations. In general, people assuming a diet rich in animal proteins, simple sugars and fats have a reduced diversity of gut microbiota with a prevalence of *Bacteroidetes* spp. Dietary intervention, fish oil, reduction in animal protein intake among others, can modify bacterial diversity in the gut sometimes leading to a mild reduction of joint inflammation. Smoking can cause several immune dysregulation and represents a risk factor for arthritis onset; in addition smoking can also affect arthritis outcome, by interfering with therapeutic response to synthetic and biological drugs. Smoking influences both oral and gut microflora with an increase in *P. gingivalis, Tannerella* and *Bacteroidetes* that can be partially reverted after smoking cessation. It is known that stress can influence the immune function and consequently autoimmune diseases via different serological and cellular pathways; a clinical role for stress in the onset and flares of patients with chronic arthritis has been reported, however it needs to be better elucidated. The axis between brain and immune system involves also the microbiota as supported by studies using several animal models and by the recent description of gut microbiota alterations in patients with depression and irritable bowel syndrome.

To identify individual parameters able to predict disease severity and therapeutic outcome in RA and SpA patients is still an unmet priority for the rheumatologist. A better elucidation of the potential role of the individual microbiota in these diseases could be a promising tool that deserves to be explored. Further studies are needed to better clarify how the changes in gut composition/diversity induced by environmental factors can reflect in the autoimmune arthritis development and prognosis however current knowledge strongly suggests to include smoking cessation and dietary intervention in the management of autoimmune arthritis patients.

## Abbreviations

SpA: spondyloarthritis
RA: rheumatoid arthritis
eRA: early rheumatoid arthritis
ILF: isolated lymphoid follicles
SFB: segmented filamentous bacteria
Treg: regulatory T
Tfh: follicular T helper
SCFAs: short-chain fatty acids
MHC: major histocompatibility complex
AS: ankylosing spondylitis
PsA: psoriatic arthritis
TLR: toll like receptors
CARD9: caspase recruitment domain-containing 9
IBD: inflammatory bowel disease
ANKENT: ankylosing enthesopathy mouse
PBMC: peripheral blood mononuclear cells
ACPAs: anti-citrullinated protein antibodies
MGWAS: metagenome-wide association study
PD: periodontitis
AIA: Ag-induced arthritis
PAD: peptidyl-arginine-deiminases
DMARDs: disease modifying antirheumatic drugs
RCTs: randomized clinical trials
PUFA: fatty acid
ARA: arachidonic acid
EPA: eicosapentaenoic acid
DHA: docosahexaenoic acid
CRP: C reactive protein
DAS28: disease activity score on 28 joints
BASDAI: bath ankylosing disease activity index
UC: ulcerative colitis
BASFI: bath ankylosing functional index
MASES: maastricht spondylitis enthesitis score
JIA: juvenile idiopatihc arthritis
SSA: sulphasalazine
CD: crohn disease
